# Orally administered β-glucan attenuates the Th2 response in a model of airway hypersensitivity

**DOI:** 10.1186/s40064-016-2501-1

**Published:** 2016-06-21

**Authors:** Ashley R. Burg, Laura Quigley, Adam V. Jones, Geraldine M. O’Connor, Kimberly Boelte, Daniel W. McVicar, Selinda J. Orr

**Affiliations:** Cancer and Inflammation Program, National Cancer Institute-Frederick, Frederick, MD 21702 USA; Department of Microbiology, University of Alabama at Birmingham, Birmingham, AL 35205 USA; University Dental Hospital, Cardiff and Vale University Health Board, Cardiff, CF14 4XY Wales, UK; Department of Biological Sciences, University of Chester, Chester, CH1 4BJ Wales, UK; Division of Infection and Immunity, Cardiff University School of Medicine, Tenovus Building, Heath Park, Cardiff, CF14 4XN Wales, UK

**Keywords:** β-Glucan, Th2, Airway inflammation, Eosinophils

## Abstract

β-Glucan is a polysaccharide that can be extracted from fungal cell walls. Wellmune WGP^®^, a preparation of β-1,3/1,6-glucans, is a dietary supplement that has immunomodulating properties. Here we investigated the effect WGP had on a mouse model of asthma. OVA-induced asthma in mice is characterized by infiltration of eosinophils into the lung, production of Th2 cytokines and IgE. Daily oral administration of WGP (400 µg) significantly reduced the influx of eosinophils into the lungs of OVA-challenged mice compared to control mice. In addition, WGP inhibited pulmonary production of Th2 cytokines (IL-4, IL-5, IL-13), however serum IgE levels were unaffected by WGP treatment. These data indicate that WGP could potentially be useful as an oral supplement for some asthma patients, however, it would need to be combined with therapies that target other aspects of the disease such as IgE levels. As such, further studies that examine the potential of WGP in combination with other therapies should be explored.

**Electronic supplementary material:**

The online version of this article (doi:10.1186/s40064-016-2501-1) contains supplementary material, which is available to authorized users.

## Background

Asthma is a chronic inflammatory airway disease that is caused by an allergic response in many cases. It affects 5–10 % of the population and is associated with significant morbidity and mortality (Barnes 2008). It is characterized by airway inflammation, airway hyperresponsiveness (AHR) and mucus production (Holgate 2012). Lung inflammation is caused by infiltration of eosinophils and T cells secreting Th2-cytokines (IL-4, IL-5, IL-13) into the lung. IL-4, IL-5 and IL-13 are important for IgE production, eosinophil recruitment and survival, mucus secretion and AHR development (Lloyd and Hessel 2010). In addition to eosinophilic Th2-associated asthma, other asthma phenotypes occur such as neutrophilic non-Th2 associated asthma. Currently the main treatment for asthma is corticosteroid administration, however some asthma phenotypes are refractory to corticosteroid therapy (Wenzel 2012). There is currently a significant level of research into the development of immunotherapies for asthma. These involve the targeting of IgE or cytokines such as IL-4, IL-5, IL-13. While many of these therapies are ineffective individually, researchers are currently examining the potential for combination therapies (Akdis 2012).

β-Glucan, a fungal cell wall extract, is marketed as a dietary supplement that can promote immune balance (Wichers 2009). β-Glucans are found in the cell walls of fungi, plants and some bacteria. β(1-3)- or β(1-6)-linked glucans are recognized by the Dectin-1 receptor. Dectin 1 is a type II transmembrane C-type lectin-like receptor, which recognizes both soluble and particulate β-glucans (Brown 2006; Brown et al. 2002, 2003). While Dectin-1 can bind both soluble and particulate β-glucans, only particulate β-glucans activate Dectin-1 signaling and responses (Goodridge et al. 2011). β-Glucan-induced signaling through Dectin-1 promotes Th1 and Th17 responses (Leibundgut-Landmann et al. 2008).

β-Glucan has been shown to exert beneficial therapeutic effects against various diseases. It has been shown to stimulate tumoricidal activity when co-administered with anti-tumor antibodies (Harada et al. 2003; Yan et al. 2005; Baran et al. 2007; Li et al. 2010; Hong et al. 2003, 2004). However, divergent results regarding the effect of β-glucan on respiratory health have been reported. It has been suggested that exposure to microbial products or mold can promote the development of asthma (Douwes 2005), while recent data suggest that β-glucans and other microbial signals may play a protective role against the development of asthma (Heederik and von Mutius 2012). Furthermore, β-glucan administration has been shown to prevent/improve symptoms of allergic rhinitis and upper respiratory tract infections (Fuller et al. 2012; Yamada et al. 2007). However, there is minimal data on the effect of β-glucan on asthma severity (Sarinho et al. 2009). In this study, we therefore examined the effect of oral administration of particulate β-glucan (WGP) in a mouse model of ovalbumin (OVA)-induced asthma. Here we demonstrate that oral administration of WGP reduces eosinophil influx and the production of Th2 cytokines (IL-4, IL-5, IL-13) in the lungs of OVA-challenged mice.

## Methods

### Animals

C57BL/6 mice were maintained under specific pathogen-free conditions at the NCI–Frederick, MD. Animal care was provided in accordance with the procedures in, “A Guide for the Care and Use of Laboratory Animals”. Ethical approval for the animal experiments detailed in this manuscript was received from the Institutional Animal Care and Use Committee at the NCI-Frederick.

### Oral administration of PBS or WGP

Highly purified particulate β-glucan WGP was isolated from the cell wall of Saccharomyces cerevisiae (Biothera, Eagen, MN). The β-glucan preparation contained 81 % β-1,3/1,6-glucan, 0.5 % mannose, <3.5 % protein and <10 % fat. Mice were treated daily with 25–100 μl PBS containing 400 μg Wellmune WGP from Biothera or PBS alone by oral gavage or alternatively by feeding by pipet as needed. Treatment was started 7 days prior to beginning OVA sensitization, and was continued for the duration of the study.

### Sensitization and airway challenge

**Fig. 1:**
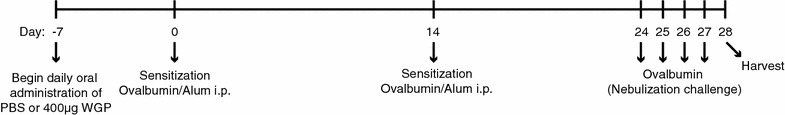
Schematic for induction of airway inflammation and treatment with WGP. PBS or 400 μg WGP in PBS was orally administered to mice daily via oral gavage or pipette feeding. Oral administration of PBS or WGP began 7 days prior to sensitization with ovalbumin (OVA)/Alum and continued daily for the duration of the experiment. Mice were sensitized via i.p. injection of 100 μg OVA in 1.8–2.6 mg Alum on Days 0 and 14. Mice were challenged with OVA (1 % w/v in PBS) for 20 min using a nebulizer on Days 24, 25, 26 and 27. Mice were euthanized 24 h following the last challenge

Allergic airway hypersensitivity to ovalbumin (OVA) was induced using grade V chicken egg OVA (Sigma, St. Louis, MO). Sensitization was initiated by an intraperitoneal (i.p.) injection of 100 μg OVA in 200 μl of 11–13 mg/ml aluminum hydroxide colloidal suspension (Alum), followed by a second i.p. injection 2 weeks later. Ten days after the last sensitization, airway hypersensitivity was induced by 20-min nebulization sessions challenging with 1 % (w/v) OVA in PBS for four consecutive days. Mice were sacrificed, and tissues harvested 24 h after the last nebulization. A schematic of the treatment schedule is shown in Fig. 1.

### Bronchoalveolar lavage fluid (BALF) collection

Prior to BALF collection, mice were given a ketamine/xylazine mixture to induce deep anesthesia and analgesia. Once sedated, as determined by toe-pinch, the thoracic cavity was opened to reveal the lungs and trachea. Using a cannula and leur lock syringe, the lungs were expanded using 1 mL of PBS and recollected as BALF, for a total of 2–3 washes. The mice were euthanized by heart perfusion using 5–10 mL PBS, and the lungs and other tissues were collected.

### BALF cytospin and differential cell count analysis

Cells from the BAL were collected, and the BALF was saved for cytokine analysis. Red blood cell lysis was performed if necessary, cells were washed in PBS, and then counted using a hemacytometer. 100 μL containing approximately 5 × 10^4^ cells were loaded onto a cytospin column and centrifuged onto a microscope slide. Following Diffquick cell staining, differential cell counts were blindly assessed for each sample, counting at least 500 cells per slide.

### BALF assays

BALF was clarified by centrifugation, aliquoted and stored at −80 °C for later analysis. Cytokine levels were measured in BALF using ELISA kits from eBioscience (San Diego, CA) and R&D Systems (Minneapolis, MN). Myeloperoxidase (MPO) activity in BALF samples was measured using the Myeloperoxidase activity fluorometric assay kit from BioVision (Mountain View, CA). Eosinophil peroxidase (EPO) in BALF samples was measured using an Eosinophil peroxidase ELISA kit (Cusabio Biotech, Wuhan, China). Soluble collagen levels in BALF were measured by adding 200 µL of 0.1 % Direct Red 80 in picric acid (Sigma Aldrich, St. Louis, MO) to 50 µL of lavage fluid and incubating for 60 min at 37 °C. The samples were centrifuged and the pellet was washed with 100 % ethanol. The pellet was resuspended in 200 µl 0.5 M NaOH, incubated for 30 min at 37 °C and the absorbance was read at 540 nm.

### Lung and spleen cell isolation and flow cytometry

Lung cells were isolated by collagenase and DNase digestion (Sigma Aldrich). Splenocytes were mashed through 100 µm nylon cell strainers and erythrocytes were lysed in ACK buffer. Lung cells were washed with Hank’s Balanced Salt Solution (HBSS) and splenocytes were washed with PBS. Lung cells and splenocytes were pre-incubated with the 2.4 G2 antibody to block Fc receptor binding, followed by incubation with various cell surface Abs: CD3 (17-A2), NK1.1 (PK136), B220 (RA3-6B2), F4/80 (BM8), MHCII (M5/114.15.2), (eBioscience, San Diego, CA), Siglec F (E50-2440), CD11c (N418), CD11b (M1/70), Gr-1 (RB6-8C5) and Ly6G (1A8) (BD Biosciences, San Jose, CA). Cells were fixed with BD Cytofix and analysed on BD LSRII.

### Blood cell measurement

Blood samples were taken prior to the start of the experiment, and again on the day of harvest. Cell composition was measured using the Hemavet 950 automated blood cell counter, (Drew Scientific, Waterbury, CT), according to the manufacturer’s protocol.

### RNA isolation and analysis

A portion of the lung was excised and placed in Trizol for RNA isolation. The lung tissue in Trizol was homogenized using a mini-beadbeater. RNA was extracted using the Trizol protocol and purified using the RNeasy Mini Kit (Qiagen, Valencia, CA). cDNA was synthesized using Taqman Reverse Transcription Reagents for RT-PCR (Applied Biosystems). Quantitative RT-PCR was performed using ABI Taqman Primer and Probe sets and normalization was performed against *Hprt1*.

### Measurement of IgE

Total IgE levels in serum were measured using the IgE ELISA from BD Biosciences (San Jose, CA). OVA specific IgE was measured using the Legend Max Mouse OVA Specific IgE ELISA kit from Biolegend (San Diego, CA).

### Lung histology

The lungs were placed in 10 % formalin, embedded in paraffin wax blocks and 4 μm sections cut and stained for either haematoxylin and eosin (H&E) or periodic acid Schiff (PAS) to determine airway inflammation and goblet cell metaplasia respectively. All histological sections were blindly assessed by a pathologist and the total number of bronchiole and perivascular aggregates of inflammatory cells were counted in each lung. Mucous cell metaplasia within the bronchioles was compared to normal control tissue and graded as 0 = no difference, 1 = scattered mucous cells, 2 = aggregates of mucous cells, 3 = monolayer layer of mucous cells and 4 = multilayered mucous cells.

### Statistical analysis

Data are expressed as mean ± SEM. Statistical significance was determined using Student’s *t* test or 1-way ANOVA followed by Bonferroni’s post-test. Statistical significance was set at *p < 0.05, **p < 0.01, ***p < 0.001.

## Results

### Systemic impact of oral WGP administration

**Fig. 2:**
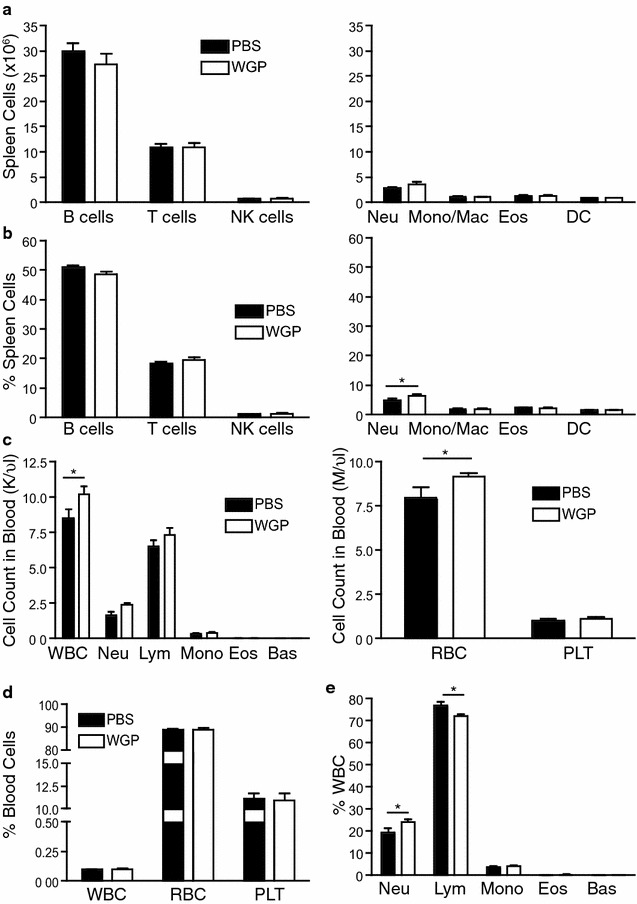
Systemic impact of oral WGP administration. Cell numbers (**a**) and percentages (**b**) in spleens from OVA-challenged mice fed with PBS or WGP. Graph displays mean ± SEM from 3 to 4 independent experiments, n = 14–19/group. Cell numbers (**c**) and percentages (**d**, **e**) in blood from OVA-challenged mice fed with PBS or WGP. Graph displays mean ± SEM from 2 independent experiments, n = 8–10/group. *p < 0.05 (1-way ANOVA, Bonferroni’s post-test)

To examine the effect of orally administered WGP on allergic airway inflammation, we sensitized and challenged WT mice with OVA alongside daily treatments of either WGP or PBS, as a vehicle control (Fig. 1). Based on previous studies where a daily dose of 400 μg of WGP was found to be the most effective dose for antitumor responses (Li et al. 2010), we administered 400 μg WGP in our study. We first examined whether WGP had any systemic effect on cellular composition in the blood or other organs as it was administered orally to the mice. Overall WGP administration did not have a major impact on cellular composition of the spleen or blood, however subtle effects on some cell populations were observed. WGP administration did not significantly affect the total number of neutrophils (Fig. 2a, c), however it modestly increased the percentage of neutrophils in both the spleen and blood (Fig. 2b, e). The WBC count was higher in WGP-treated mice, while the percentage of WBC was not affected (Fig. 2c, d). The WGP-treated mice displayed a small decrease in the percentage of lymphocytes, corresponding to the increase in the percentage of neutrophils in the blood (Fig. 2c, e). Interestingly, other populations including eosinophils and monocytes/macrophages in the spleen and the blood were unaffected by oral administration of WGP.

### WGP reduces total BALF cell numbers and eosinophil accumulation

**Fig. 3:**
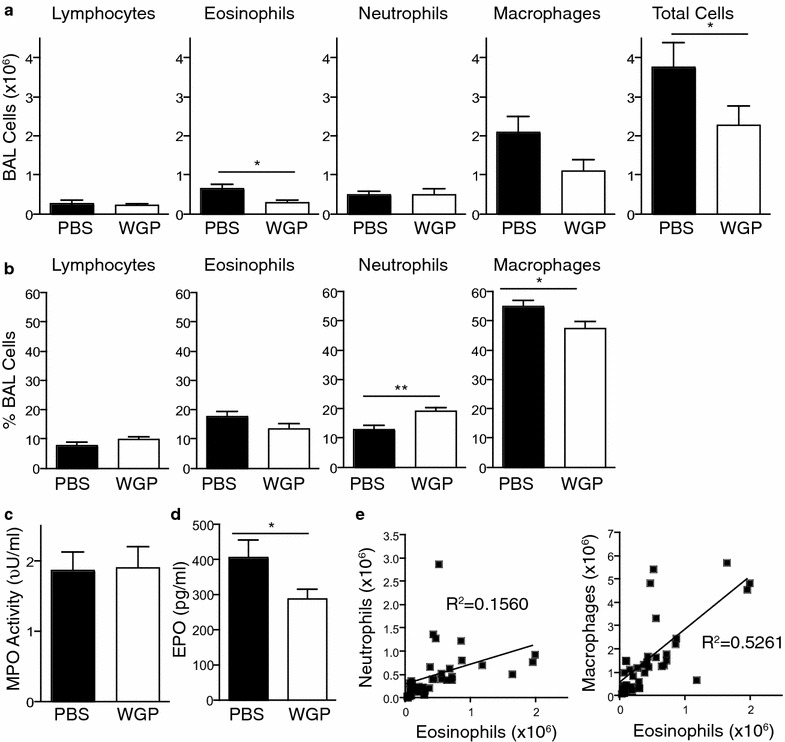
Effect of oral administration of WGP on BALF cells in OVA-challenged mice. Cell numbers (**a**) and percentages (**b**) in BALFs from OVA-challenged mice fed with PBS or WGP. Graphs display mean ± SEM from 4 independent experiments, n = 19/group. **c** MPO Activity in BALF. Graph displays mean ± SEM from 5 independent experiments (n = 21–22/group). **d** EPO levels in BALF. Graph displays mean ± SEM from 3 independent experiments (n = 15/group). **a**–**d** *p < 0.05 (Student’s *t* test). **e** Graphs display correlation between number of eosinophils and number of neutrophils or macrophages present in BALF

We next examined the effect of orally administered WGP on the composition of cell populations present in the lungs of mice challenged with OVA. To this end, BALF was collected from the lungs and analysis of cells recovered from the BALF revealed that oral administration of WGP significantly changed the composition of inflammatory cells accumulating due to OVA-induced airway hypersensitivity. The total number of cells, total number of eosinophils and the percentage of macrophages were significantly reduced while the percentage of neutrophils was increased in the WGP-treated mice (Fig. 3a, b). As MPO activity and EPO levels correspond to the number of neutrophils and eosinophils, respectively in a sample, we next measured MPO and EPO levels in the BALF. While the percentage of neutrophils was increased in WGP-treated mice, MPO activity in the BALF was similar between PBS and WGP-treated mice (Fig. 3c). This is likely due to the similar total number of neutrophils in the BALF from PBS and WGP-treated mice. EPO levels were reduced in WGP-treated mice (Fig. 3d), which is consistent with the reduced eosinophil numbers in the BALF from these mice. Correlation analysis was then performed to determine whether there was a link between the number of eosinophils and the number of neutrophils and/or macrophages in the BALF. While there was no correlation between the number of eosinophils and neutrophils, a positive correlation between the number of eosinophils and macrophages was observed (Fig. 3e). These data indicate that oral administration of WGP significantly inhibits the local recruitment of inflammatory cells, in particular eosinophils and to a lesser extent macrophages, to the lungs in this model of allergic inflammation while it promotes a subtle systemic increase in neutrophils.

### WGP modulates lung cellular composition

**Fig. 4:**
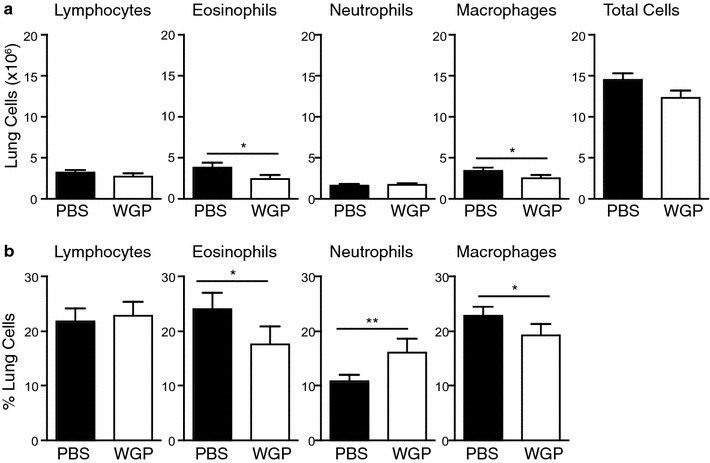
Effect of oral administration of WGP on lung cellular composition in OVA-challenged mice. Cell numbers (**a**) and percentages (**b**) in lungs from OVA-challenged mice fed with PBS or WGP. Graphs display mean ± SEM from 3 independent experiments, n = 13–14/group. **a**–**d** *p < 0.05, **p < 0.01 (Student’s *t* test)

As we observed differences in BALF cellular composition, we examined the effect of WGP on lung cellular composition. Similar to our findings in BALF, infiltration of both eosinophils and macrophages were significantly reduced in both percentage (Fig. 4a; Additional file 1: Fig. S1) and total cell number (Fig. 4b). While the percentage of neutrophils was increased in the lungs of WGP-treated mice compared to PBS-treated mice (Fig. 4b; Additional file 1: Fig. S1), no significant difference was observed in the total number of infiltrating neutrophils (Fig. 4a), nor were there any alterations in lymphocyte infiltration (Fig. 4a, b). Taken together these data indicate that WGP significantly inhibits the recruitment of eosinophils and macrophages to the lungs of these mice while it promotes a modest increase in neutrophil recruitment.

### WGP does not affect OVA-induced histopathological changes in the lung

**Fig. 5:**
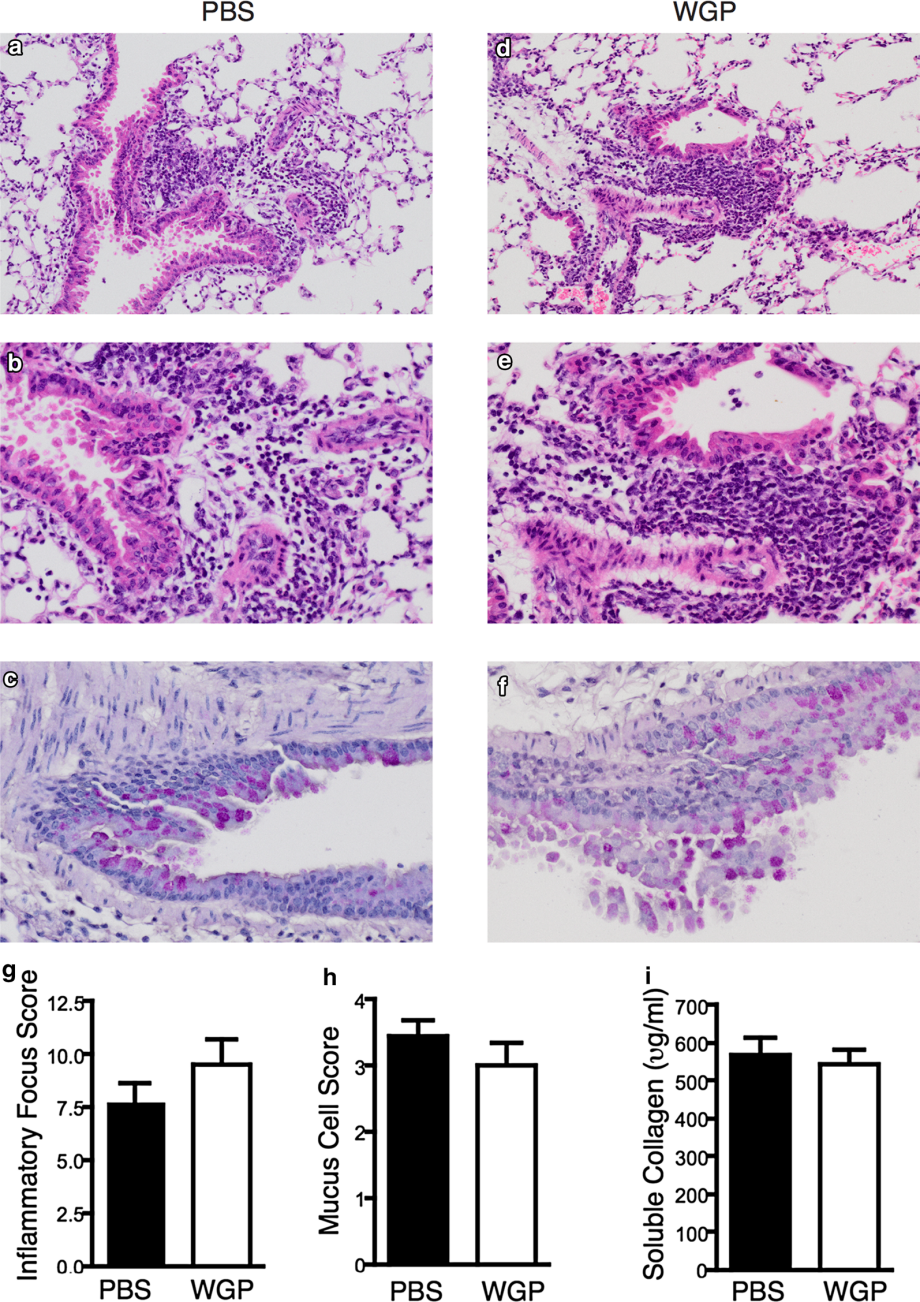
Effect of oral administration of WGP on lung histology and soluble collagen. **a**–**f** Representative sections from both treatment groups demonstrate the varied morphological changes seen within the lung. H&E sections from a representative mouse treated with PBS (**a**, **b**) and a representative mouse treated with WGP (**d**, **e**). PAS stains from different areas reveal mucous cell metaplasia in each group (**c**, **f**). **g**, **h** Graphs display mean ± SEM. Inflammatory Focus Score (**g**) and Mucus Cell Score (**h**). Graphs are the cumulative result of 2 independent experiments (n = 10). **i** Graph displays mean ± SEM of soluble collagen levels in BALF. Graph is the cumulative result of 5 independent experiments (n = 22–23)

To determine the effect of WGP on OVA-induced histopathological changes in the lung, lung sections were stained with H&E and scored. H&E sections from a representative mouse treated with PBS (Fig. 5a, b) demonstrate the expected prominent epithelial infolding and lumen reduction related to mucous cell metaplasia associated with OVA-induced inflammatory response. The surrounding tissue shows peribronchiole and perivascular inflammatory infiltrates comprising lymphocytes with scattered eosinophils. Similar features were seen in mice treated with WGP (Fig. 5d, e) although in this section the changes within the bronchiole are subtle and the lymphocytic infiltrate denser. Variable changes were seen in different areas of the lung with a mixed inflammatory cell infiltrate predominantly observed in peribronchial and perivascular areas in OVA-challenged mice; however, WGP had no effect on these changes (Fig. 5a, b, d, e, g). Similar features were seen in bronchioles of both PBS and WGP-treated mice with folded epithelium and narrowing of lumens and no differences in goblet cell metaplasia or mucous production (Fig. 5c, f, h). As lung fibrosis/airway remodeling is characterized by enhanced collagen deposition (Yamauchi et al. 2008) we assayed the BALF for soluble collagen. Similar to the histological findings, soluble collagen levels in the lung were similar between PBS and WGP-treated mice (Fig. 5i), demonstrating that some aspects of the airway hyperesponsiveness model are unaffected by WGP treatment.

### WGP reduces Th2 cytokine levels in the lung

**Fig. 6:**
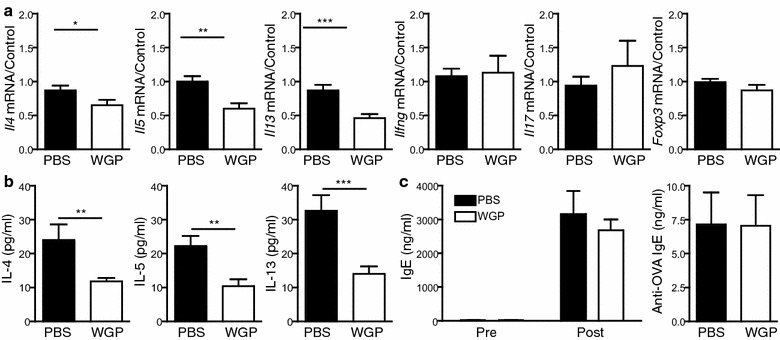
Cytokine levels and IgE levels in OVA-challenged mice. **a** RNA was extracted from lung tissue from OVA-challenged mice fed with PBS or WGP. *Il4, Il5, Il13, Ifng, Il17* and *Foxp3* mRNA levels were analyzed by Real Time qPCR. Graphs display mean ± SEM from 3 independent experiments, n = 14–15/group. **b** Cytokine levels were measured in BALF by ELISA. Graphs display mean ± SEM from 3 independent experiments, n=12–15/group. **a**–**b** *p < 0.05, **p < 0.01, ***p < 0.001 (Student's *t* test). **c** Total IgE and OVA-specific IgE levels were measured in the serum by ELISA. Graph displays mean ± SEM from 5 independent experiments, n=23–25/group

Th2 cells secreting IL-4, IL-5 and IL-13 are important for eosinophil recruitment and survival and for IgE production (Monick et al. 2001). To further investigate the effect of WGP on allergic inflammation, cytokine levels (Th1, Th2 and Th17) in the lungs of PBS and OVA-challenged mice were measured. Consistent with previously published data from this model, OVA challenged mice displayed increased levels of these cytokines in the lungs compared to PBS challenged mice (data not shown). Interestingly, the levels of *Il4* and *Il5* mRNA, which are important for eosinophil recruitment, along with *Il13*, were reduced in the lungs of WGP-treated OVA-challenged mice (Fig. 6a), while Th1 (*Ifng*) and Th17 (*Il17*) cytokine mRNA levels remained similar between PBS and WGP-treated OVA-challenged mice. In addition there was no change in the Treg population, as mRNA levels of *Foxp3*, the transcription factor for Tregs, were similar between PBS and WGP-treated mice (Fig. 6a). Consistent with the mRNA data, IL-4, IL-5 and IL-13 protein levels were significantly reduced in WGP-treated mice while IFN-γ and IL-17 levels were mostly not detected (Fig. 6b and data not shown). As IgE production is an important aspect of the OVA-challenge asthma model, we next examined IgE production in the serum of these mice. Total IgE levels and OVA-specific IgE levels (Fig. 6c) were similar between PBS and WGP-treated mice. Together, these data demonstrate reduced Th2 cytokine production in WGP-treated mice compared to PBS-treated mice while Th1 and Th17 cytokine levels are similar between the two groups.

## Discussion

Several natural products are advertised as immunotherapeutics and many claims are made about their effects on a wide range of diseases including asthma. Here we tested the effect of Wellmune WGP β-1,3/1,6-glucan during a mouse model of OVA-induced asthma. We demonstrated that daily oral administration of WGP reduces eosinophil influx into the lungs and the production of Th2 cytokines (IL-4, IL-5, IL-13) compared to control mice. Serum IgE levels and OVA-specific IgE levels were unaffected by administration of WGP. Together these data indicate that WGP administration would not, in and of itself, be sufficient as a single treatment for asthma, however as it targets the critical initiator step in asthma immune responses (or similar) it may prove useful as a combination therapy.

Asthma is a heterogeneous disease and patients present with various distinct clinical phenotypes. Different mouse models are being developed to model the different phenotypes found in asthma patients (Lloyd and Hessel 2010; Nials and Uddin 2008; Lloyd 2007). The OVA-induced airway hypersensitivity model of asthma used here results in eosinophilic inflammation in the lung, pulmonary production of Th2 cytokines (IL-4, IL-5 and IL-13) and serum IgE production (Lloyd and Hessel 2010; Lloyd 2007). The role of Th2 cells in patients with asthma is well established and T cell activation has been related to asthma severity in some studies (Walker et al. 1991; Robinson et al. 1993). IL-4, IL-5 and IL-13 have been shown to have roles in IgE production, eosinophil survival, AHR development and tissue remodeling (Finkelman et al. 2010). Here we observed a significant reduction in production of Th2 cytokines and eosinophil influx with WGP administration, although this does not reach a threshold to see histological differences. Additionally, IgE production and soluble collagen levels were unaffected by the administration of WGP. Therefore, while other factors in addition to Th2 cells/cytokines are involved in the pathogenesis of asthma (Lloyd and Hessel 2010), it appears that WGP is specific in targeting the production of IL-4, IL-5, IL-13 and resulting eosinophilia in this model. This targeted effect is important as local production of these cytokines is associated with AHR development and downstream complications. While our results indicate no impact on collagen-deposition or lung pathology, these studies may only represent an acute effect. It is possible that with long-term WGP-administration after asthma is established or in combination with other therapies, the continued decrease in Th2 responses could protect against damaging inflammation and fibrosis in the lung.

β-Glucan has immunomodulating properties and it has been shown to exert beneficial therapeutic effects against various diseases including allergic diseases. One study demonstrated that administration of WGP to ragweed allergy sufferers reduced allergy symptoms however similar to our findings it had no effect on serum IgE levels (Talbott et al. 2013). Another study showed that β-glucan administration to subjects with seasonal allergic rhinitis resulted in reduced IL-4 and IL-5 levels (Kirmaz et al. 2005). Our data demonstrating reduced IL-4 and IL-5 levels in WGP-treated mice in an OVA-induced asthma model is in agreement with these findings. In addition a study demonstrated a reduction in asthmatic symptoms following β-glucan administration and increased IL-10 levels (Sarinho et al. 2009).

## Conclusions

Daily oral administration of WGP (400 µg) significantly reduced pulmonary eosinophil influx and production of Th2 cytokines (IL-4, IL-5, IL-13), however serum IgE levels were unaffected by WGP treatment in an OVA-induced asthma model. Our findings suggest that WGP could be used to target some aspects of asthma (Th2 cytokines and eosinophilia). While WGP alone would not be sufficient to treat asthma, it could be used in combination with other therapies that target different aspects of the disease such as IgE levels. As WGP is an oral supplement, it could be considerably more cost effective and may have fewer side effects than immunotherapies. In addition, as IL-4, IL-5 and IL-13 are all reduced in response to WGP, the broader effects of WGP could be more beneficial than immunotherapies that target one specific cytokine such as IL-4 or IL-5. Further studies would need to be conducted to determine the effect of WGP in combination with other therapies for specific subsets of asthma patients.

## Additional file

10.1186/s40064-016-2501-1 Effect of oral administration of WGP on lung cellular composition in OVA-challenged mice. Representative flow cytometry plots of lymphocyte (A), eosinophil (B), neutrophil and macrophage (C) percentages in isolated lung cells from OVA-challenged mice fed with PBS or WGP.
